# Prevalence of Feeding and Swallowing Disorders in Congenital Heart Disease: A Scoping Review

**DOI:** 10.3389/fped.2022.843023

**Published:** 2022-04-05

**Authors:** Vivienne Norman, Liesl Zühlke, Katherine Murray, Brenda Morrow

**Affiliations:** ^1^Division of Communication Sciences and Disorders, Department of Health and Rehabilitation Sciences, University of Cape Town, Cape Town, South Africa; ^2^Department of Paediatrics and Child Health, University of Cape Town, Cape Town, South Africa; ^3^Division of Paediatric Cardiology, Department of Paediatrics and Child Health, Red Cross Children's Hospital, University of Cape Town, Cape Town, South Africa; ^4^Division of Cardiology, Department of Medicine, Groote Schuur Hospital, University of Cape Town, Cape Town, South Africa

**Keywords:** aspiration, dysphagia, swallowing, infants, congenital heart disease

## Abstract

Feeding and swallowing difficulties are commonly reported as comorbidities in infants and children with congenital heart disease. These difficulties have negative health consequences for the child and impact the quality of life of both the child and caregivers. This scoping review presents an integrated summary of the published literature on the prevalence of feeding and swallowing difficulties in congenital heart disease. Fifteen peer-reviewed articles, written in English and published in the last 25 years, were included in the review, following a search of relevant databases. The studies reported on a total of 1,107 participants across the articles ranging in age from premature infants to children aged 17 years. An overall pooled prevalence of 42.9% feeding and swallowing difficulties was reported, with a prevalence of 32.9% reporting aspiration. A wide prevalence range of feeding and swallowing difficulties was reported across the articles and factors that contributed to this included the ages of participants, and the definition and assessment of feeding and swallowing difficulties used in the studies. The review confirms that feeding and swallowing difficulties are common in infants and children with congenital heart defects, and that assessment and management of these difficulties should be considered part of the standard of care.

## Introduction

Congenital heart disease (CHD) is the most common congenital abnormality, affecting ~9 infants per 1,000 live births (1). These abnormalities range from mild heart defects to complex lesions such as critical single ventricle abnormalities, for which infants will likely require intervention and extended periods of hospitalization (2), including post-surgical and unscheduled admissions to the pediatric intensive care unit (PICU).

Feeding and swallowing difficulties (FSD) are common in infants with CHD and associated with significant morbidity, including respiratory disease, poor weight gain, longer duration of PICU and hospital stays, and increased caregiver stress (3). In addition, poor oral feeding is a common reason for delaying discharge from hospital (4).

There are several factors associated with FSD in infants and young children with CHD, including difficulty coordinating breathing and swallowing due to increased respiratory rate and effort of breathing associated with CHD, and fatigue and reduced endurance resulting in inadequate caloric intake (5). Prolonged enteral feeding due to fragility and increased nutritional requirements may result in a lack of exposure to oral feeds and, therefore, difficulties or delays in the development of feeding skills. Furthermore, gastro-esophageal reflux is common in infants with CHD and frequently associated with FSD, and an increased risk of aspiration has been noted (3). Vocal cord dysfunction, which may be associated with recurrent laryngeal nerve injury after cardiac surgery, has been reported to increase the risk of aspiration when swallowing because of inadequate airway closure and protection (6).

The purpose of this scoping review was to summarize, integrate and interpret literature on the prevalence of FSD (oropharyngeal dysphagia) in infants and children with CHD. This information can be used to identify gaps in research and inform clinicians about the potential risk of FSD in infants and children with CHD. Earlier identification of FSD and referral for appropriate management may then be implemented, ultimately reducing the negative sequelae of FSD.

## Materials and Methods

### Identifying the Research Question

The methodological framework proposed by Arksey and O'Malley (7) was used for the scoping review. This framework includes five steps, namely: (1) identifying the research question; (2) identifying relevant studies; (3) study selection; (4) charting the data; and (5) collecting, summarizing and reporting results.

The research question was “What is the prevalence of FSDs in infants and children with CHD?”

### Identifying Relevant Studies

Studies were identified by searching the following electronic databases: PubMed, Medline, Scopus, EBSCOhost, Web of Science, Cumulative Index to Nursing and Allied Health (CINAHL) and the Cochrane Library. Keywords and search terms were selected for the initial search and refined during the search process to ensure that all possible articles were included for review. The reference lists of articles identified in the searches were also reviewed to identify any additional articles for inclusion.

Combinations of keywords and/or Medical Subject Headings (MeSH) were used and adapted to meet the criteria of the relevant databases (see Supplementary Table 1 for an example of the full electronic search strategy).

### Study Selection

Only peer-reviewed articles published in English in the last 25 years (1995–2020) that reported on FSD in the pediatric population (0–18 years) with CHD were included in the scoping review. All study designs, except reviews of other studies, were included. Gray literature was not included.

One author (VN) conducted the searches and initial exclusion at title and abstract level. After the abstract level exclusion, 22 articles remained and the full text of these 22 articles was reviewed independently by VN and KM who determined the final selection of 15 articles with 100% agreement.

### Charting the Data

A standardized data extraction form was used to extract data from the included articles.

### Analysis

The data regarding prevalence of FSD were analyzed descriptively and are presented as proportions of the study population with confidence intervals. The confidence intervals were calculated using the sample proportion with FSD, the total sample, a confidence level of 95% and a z-value of 1.96 (8). The pooled prevalence of FSD was also determined for the studies.

The quality of the studies was assessed using an adapted Newcastle-Ottawa Scale (NOS) with a maximum score of 7 points (see Supplementary Table 2 for further details). One point was allocated to each of the following categories: representativeness of the sample, sample size (adequate and justified), clear definition of FSD provided, clear description of the assessment of FSD (clinical or instrumental) provided, the study controlled for other conditions associated with FSD, the study reported on assessment of outcomes, and appropriate statistical analysis was conducted and included.

**Figure 1:**
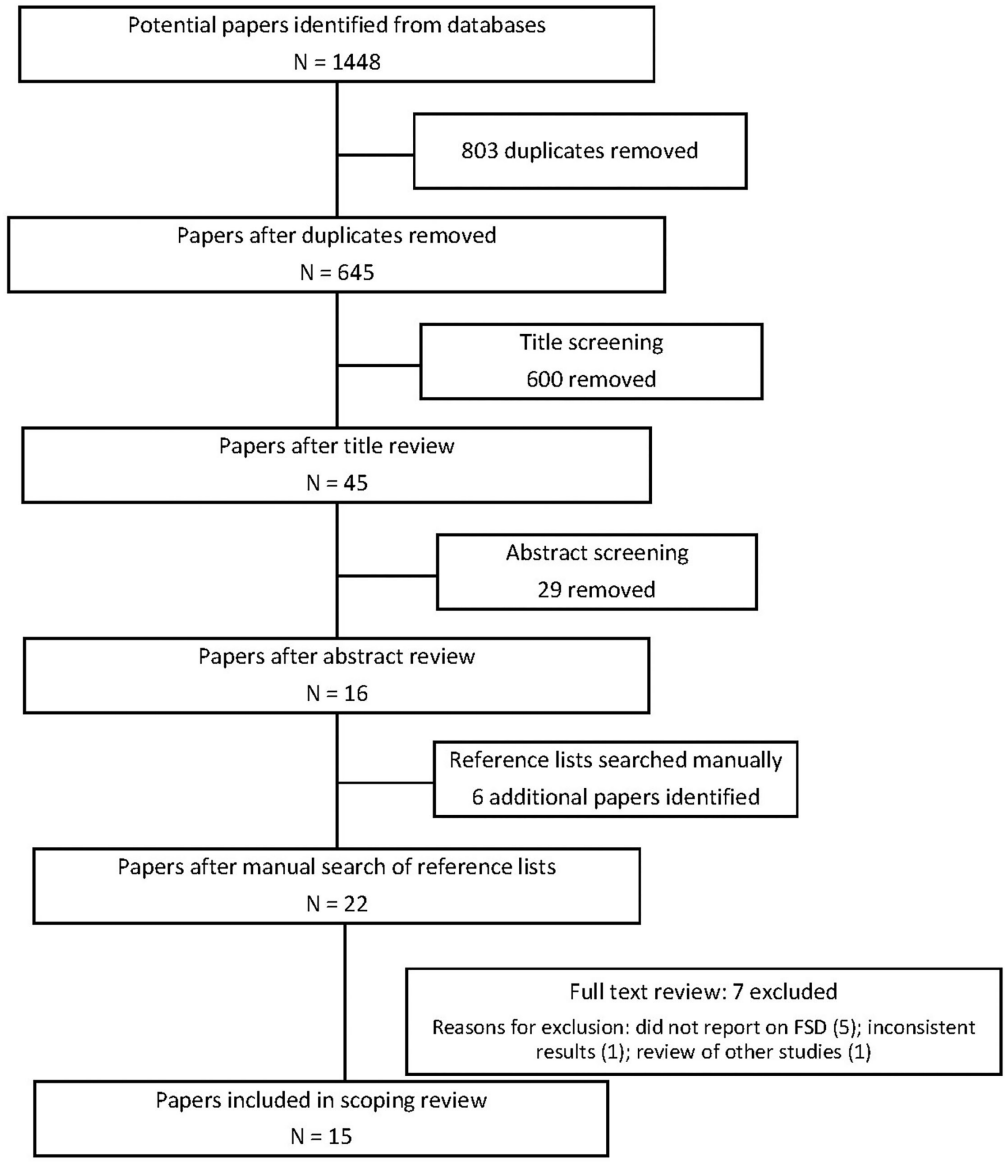
Prisma flowchart.

Figure 1 provides an overview of the review process.

## Results

Fifteen articles met the inclusion criteria of the study (see Table 1). Most studies (10/15; 67%) were published in the United States. Of the remaining five studies, four were published in other high-income countries, with one each from Australia, Canada, Korea and Switzerland. Only one study (10) was conducted in a middle-income country, namely Brazil.

**Table 1:** Summary of findings.

**Author and Title**	**Location**	**Research design**	**Sample size**	**Participant description**	**Recruitment age**	**Comparison group**	**Follow-up duration**	**Prevalence of FSD**	**Adapted NOS score**
Davis et al. (9)	US	Retrospective chart review	53	27 infants with HLHS; 26 infants with d-TGA No gestational age not reported	Birth	Compared HLHS with d-TGA	12 months	49% required some form of tube feeding at discharge: 25% NGT; 21% NGT + oral; 3% g-tube; 8% aspirated	3/7
De Souza et al. (10)	Brazil	Cross-sectional study	31	31 infants with diagnosis of CHD including: septal defects (ASD; VSD; AVSD); PDA; pulmonary stenosis; aortic supravalvular stenosis; coarctation of the aorta; TGA; tricuspid atresia; intracardiac tumor; patent foramen ovale; HLHS; aortic arch hypoplasia. No gestational age reported	13–42 days (mean 21 days)	None	None	74% dysphagia 32% (10) = mild 23% (7) = moderate 19% (6) = severe	6/7
Einarson and Arthur (11)	Canada	Retrospective chart review	101	101 neonates with CHD requiring surgical intervention in the first 28 days of life. CHD diagnoses included: univentricular heart; left-sided abnormalities; total anomalous venous drainage; TGA; TOF; truncus arteriosis. No gestational age reported	Neonates (0–28 days)	None	Until hospital discharge (up to 4 months)	28.7% non-oral feeding at discharge	5/7
Hill et al. (12)	US	Prospective cross-sectional	56	56 participants; 2–6 years with single ventricle defects; completed stage 2 palliation before age 2 years. No gestational age or history of prematurity reported.	2–6 years	Compared children with single ventricle defects to “normal population cohort”	None	28 (50%) feeding dysfunction. Caregivers of children with CHD reported significantly more of the following difficulties: “food manipulation” (*p* <0.001); “mealtime aggression” (*p* = 0.002); “choking, gagging and vomiting” (*p* <0.001); “child's resistance to eating” (*p* <0.001); and “parental aversion to mealtimes” (*p* <0.001)	3/7
Kogon et al. (13)	US	Retrospective review	83	83 participants who had surgery for CHD in first 15 days of life (neonates). Mean gestational age of 38.3 weeks ± 1.82.	Neonates (<15 days)	None	Until hospital discharge	11% required prolonged time to reach full oral feeds (>19 days) 45% discharged home with tube feeding	3/7
Kohr et al. (14)	US	Prospective, cross-sectional	50	50 participants 0–17 years evaluated post TEE. CHD included: anomalous left coronary artery; anomalous pulmonary venous drainage; aortic stenosis; ASD; VSD; cardiomyopathy; coarctation of aorta with VSD; complex single ventricle-HLHS or tricuspid atresia; congenital mitral stenosis; pulmonary atresia; Taussig-Bing anomaly; TOF. Excluded preterm infants.	0–17 years	None		18% dysphagia	6/7
Lundine et al. (15)	US	Retrospective cohort chart review	50	50 infants with single ventricle physiology who underwent hybrid procedure (and had VFSS results post-surgery). CHD of HLHS or functional single ventricle. Included premature infants; reported no statistically significant relationship between prematurity and aspiration.	Neonates	None	Unknown	44% normal; 28% penetration on Penetration-Aspiration Scale and 28% aspiration (13/14 silent aspiration).	6/7
Maurer et al. (16)	Switzerland	Retrospective study	82	82 participants at 2 years who had surgery for CHD in first 32 days of life. CHD diagnoses included: TGA; coarctation of the aorta; VSD; double outlet right ventricle with unobstructed outflow tract; TAPV; interrupted aortic arch; tricuspid atresia; pulmonary atresia and ventricular septal defect; TOF; common arterial trunk; HLHS; double inlet left ventricle with hypoplastic aortic arch; complete atrioventricular block; myocardial tumor; PDA. Included participants with a history of prematurity; at 2 years reported no association between history of prematurity and feeding difficulties.	24 months	None	None (assessed at 2 years with retrospective information)	22% feeding and swallowing difficulties at 2 years	4/7
McGrattan et al. (17)	US	Prospective cross-sectional study	36	36 infants (0–36 days) with functional single ventricles following stage 1 palliation; 24 Norwood procedure and 12 Hybrid. CHD diagnoses included: HLHS; right ventricle dominant atrioventricular septal defect; mitral and aortic stenosis; interrupted aortic arch with ventricular septal defect; double outlet right ventricle with straddling mitral valve; double inlet left ventricle with interrupted aortic arch. No gestational age reported.	Neonates (0–36 days)	Compared those who underwent Norwood procedure and Hybrid	None	83% (30) penetration on liquids 50% (18) aspiration on liquids	5/7
McKean et al. (18)	Australia	Retrospective cohort study	79	79 neonates who underwent cardiac surgery during neonatal period (with data for 3 years). CHD diagnoses included: coarctation of aorta; TGA; functional single ventricle; pulmonary atresia; HLHS; TAPV; TOF; truncus arteriosus; interrupted aortic arch. 7 of 79 participants were preterm; reported no statistically significant difference between preterm and term participants with regard to the need for a feeding tube at discharge.	Neonates (<28 days)	None	3 years	30% discharged with feeding tube	4/7
Pham et al. (19)	US	Retrospective chart review	104	104 neonates requiring Norwood procedure or aortic arch reconstruction. 7 participants were preterm; did not report on preterm participants separately.	Neonates	Compared Aortic arch reconstruction and Norwood procedure	Mean of 11.5 months (up to 72 months)	63.5% dysphagia	5/7
Pourmoghadam et al. (20)	US	Retrospective chart review	89	89 infants undergoing Norwood procedure or aortic arch repair follow-up for ± 3 years. CHD diagnoses included: HLHS; single ventricle with aortic arch hypoplasia; hypoplastic aortic arch with/without VSD; interrupted aortic arch with VSD; hypoplastic aortic arch with TGA No gestational age reported.	Neonates	Norwood procedure compared to aortic arch repair	Up to 3 years	48% (43/89) vocal cord dysfunction. 71 participants had VFSS and 42% aspirated. 53 participants had gastrostomy tube placed.	4/7
Raulston et al. (21)	US	Retrospective chart review	96	96 participants who had surgery for CHD in the first 100 days AND had FEES/MBS post-operatively before initiating oral feeds. 28 of 96 participants were preterm; reported no significant association between prematurity and aspiration.	<120 days	None	± 60 days (for some but not part of protocol)	51% aspirated on FEES or MBS	3/7
Skinner et al. (22)	US	Prospective cross-sectional study	51	51 infants with CHD, including HLHS, aortic arch hypoplasia, aortic coarctation with VSD, VSD, and coarctation with TGA. 33 underwent Norwood procedure; 18 underwent aortic arch reconstruction as part of biventricular repair. No gestational age reported.	Neonates	Compared Norwood to biventricular aortic arch repair	1 year (for some)	51% overall swallowing dysfunction 28% aspirated. Swallowing dysfunction presented in 48% following Norwood (aspiration 24%) and swallowing dysfunction in 59% following Biventricular (35% aspiration).	5/7
Yi et al. (23)	Korea	Retrospective chart review	146	146 infants (<12 months) who had cardiac surgery. CHD diagnoses included: large ventricular septal defect or double-outlet right ventricle with unobstructed outflow tract; coarctation of the aorta; TOF; HLHS; TGA; interrupted aortic arch. Mean gestational age was 38 weeks; no specific mention of prematurity.	<12 months (mean = 3.4 months)	None	Unclear. Follow- up VFSS done up to 6 months after surgery	24% (35/146) dysphagia	6/7

*HLHS, hypoplastic left heart syndrome; (d-)TGA, (dextro)-Transposition of the Great Arteries; NGT, nasogastric tube; VFSS, videofluoroscopic swallow study; TOF, Tetralogy of Fallot; CHD, congenital heart disease; FEES, fibreoptic endoscopic evaluation of swallowing; MBS, modified barium swallow; ASD, atrial septal defect; VSD, ventricular septal defect; AVSD, atrioventricular septal defect; PDA, patent ductus arteriosus; TAPVD, Total anomalous pulmonary venous drainage; VCD, vocal cord dysfunction; TEE, transesophageal echocardiography*.

*Aspiration was documented on videofluoroscopic swallow studies or fiberoptic endoscopic evaluation of swallowing; vocal cord dysfunction was assessed by laryngoscopy*.

All the studies were published in the last 17 years (from 2003 onwards) with two-thirds (67%) published in the last 10 years. Most studies (10/15; 67%) used a retrospective chart review design, while the remaining five studies (10, 12, 14, 17, 22) used prospective cross-sectional or cohort designs. The adapted NOS scores for the included studies ranged from three to six out of a maximum of seven points, with a mean of 4.5 (see Supplementary Table 2 for further details of the scoring).

A total of 1,107 participants were included across the articles, ranging in age from premature infants to children aged 17 years. Most of the studies (12/15; 80%) included neonates and infants (*n* = 919). Two studies (18, 20) reviewed data for participants recruited as neonates with follow-up to 3 years of age (*n* = 168). Hill et al. (12) included participants between the ages of 2–6 years (*n* = 56), and Kohr et al. (14) included participants aged 0–17 years (*n* = 50). Maurer et al. (16) reviewed the feeding of 2-year-old participants who had undergone surgery for CHD during the neonatal period.

Most studies (10/15; 67%) recruited participants during the neonatal period. Follow up information was reported by some authors, with variable duration, e.g., discharge from hospital (11, 13); one-year post-surgery (9, 22); 2 years of age (16); 3 years (18, 20); and up to 5 years in some participants (19). As many of the studies followed a retrospective research design, data were not available for all participants on follow-up.

All participants had a diagnosis of CHD, as described in Table 1. The majority reported on FSD in infants and children with cono-truncal abnormalities such as transposition of the great arteries, left ventricular outflow tract obstructions such as hypoplastic left heart syndrome (HLHS), anomalous pulmonary venous return, and septal defects.

Davis et al. (9) specifically compared FSD in participants with HLHS and (dextro)-Transposition of the Great Arteries (d-TGA), while Hill et al. (12) and Lundine et al. (15) both described FSD in participants with single ventricle physiology. Only post-operative feeding and swallowing difficulties were reported in all included studies. FSD in participants who underwent different surgical procedures were reported, particularly those who had a Norwood procedure (*n* = 217) (17, 19–22) and these were compared with other surgical procedures, including aortic arch reconstruction (*n* = 111) (19–22) and hybrid procedures (*n* = 12) (17). No clear statistically significant differences in FSD were reported between participants undergoing different surgical procedures.

All the studies reported on FSD, although the definitions and assessments used for the diagnosis of FSD varied greatly, making it difficult to compare the results reported across studies. The definitions, assessment, and prevalence of FSD for each article are presented in Table 2.

**Table 2:** Feeding and swallowing difficulties in congenital heart disease: definition, assessment and prevalence.

**Author**	**Definition of FSD**	**Assessment of FSD**	**Prevalence of FSD (sample size of study reported as N)**
Davis et al. (9)	No clear description/definition of dysphagia provided. Described the method of feeding at discharge.	Not described.	*N* = 53: 49% required tube feeding at discharge: 25% NGT; 21% NGT + oral; 3% g-tube. 8% aspirated
De Souza et al. (10)	Classified according to a protocol: “Classification of the Degree of Pediatric Dysphagia” which includes a range from normal, mild, moderate-severe and severe OPD with a high risk of aspiration, as assessed by an SLT.	Clinical assessment conducted by SLT	*N* = 31: 74% dysphagia: 32% (10) = mild; 23% (7) = moderate: 19% (6) = severe.
Einarson and Arthur (11)	“Infant not entirely orally fed (breast/bottle/both) at the time of discharge from hospital.”	None.	*N* = 101: 28.7% non-oral feeding at discharge.
Hill et al. (12)	Any positive subcategory on the Mealtime Behavior Questionnaire (MBQ) or About Your Child's Eating (AYCE) was considered an indication of feeding difficulty.	MBQ and AYCE questionnaires completed by caregiver.	*N* = 56: 28 (50%) feeding dysfunction.
Kogon et al. (13)	“Postoperative feeding difficulty was defined by: (1) a prolonged time to reach goal feeds; (2) a prolonged transition to oral feeds requiring tube feeds at discharge; and (3) the need for additional procedures to facilitate feeding.”	None	*N* = 83: 11% required prolonged time to reach full oral feeds (>19 days). 45% discharged home with tube feeding.
Kohr et al. (14)	Diagnosis of dysphagia made by SLT after clinical swallowing assessment.	Clinical assessment conducted by SLT	*N* = 50: Dysphagia 18%.
Lundine et al. (15)	Swallowing dysfunction described as penetration or aspiration on VFSS.	VFSS	*N* = 50: 44% normal; 28% penetration and 28% aspiration (13/14 silent aspiration) on Penetration-Aspiration Scale.
Maurer et al. (16)	“Feeding disorder was defined as the presence of one or more of the following three criteria at the age of 2 years, as judged by the primary care provider: (1) partially or completely dependent on tube feeding; (2) feeding behavior is not age-adequate, i.e., only drinks liquids or eats pureed food; (3) failure to thrive, i.e., the weight of the child is below the third percentile.”	None	*N* = 82: 22% FSD at 2 years.
McGrattan et al. (17)	A difficulty in any component noted in the evaluation of the oropharyngeal swallow on VFSS was considered a symptom of dysphagia.	VFSS	*N* = 36: 83% penetration and 50% aspiration with liquids.
McKean et al. (18)	Feeding difficulty was defined as “the requirement for ongoing tube feeding at the time of discharge home or transfer to another hospital.”	None (only 8% had VFSS)	*N* = 79: 30% discharged with feeding tube.
Pham et al. (19)	“An inability to tolerate adequate oral intake without supplementation by nasogastric (NG) tube feeding.”	Not all participants were assessed; assessments included clinical swallowing evaluation, VFSS or an upper GI study	*N* = 104: 63.5% dysphagia.
Pourmoghadam et al. (20)	No clear definition provided.	Clinical assessment by SLT and some underwent oropharyngeal motility study.	*N* = 89 but only 71 had VFSS: 42% aspiration. 48% vocal cord dysfunction. 53 participants had gastrostomy tube placed.
Raulston et al. (21)	No definition provided – assessed clinically and with FEES/VFSS to assess for aspiration.	A clinical swallowing evaluation by SLT and either FEES or VFSS.	*N* = 96: 51% had aspiration on FEES or VFSS.
Skinner et al. (22)	Definition not provided; swallowing dysfunction identified on VFSS results.	VFSS +/- laryngoscopy	*N* = 51: 52% overall swallowing dysfunction; 28% aspiration. Swallowing dysfunction presented in 48% following Norwood (24% aspiration) and in 59% following Biventricular (35% aspiration).
Yi et al. (23)	Dysphagia was defined as one of the following conditions: “(1) feeding desaturation, increased work required for breathing during feeding, coughing/choking during feeding, altered crying, or other signs; (2) failure of any clinical modification in improving oral feeding; and (3) tube feeding until discharge.”	VFSS conducted in 33 of the 35 participants diagnosed with dysphagia.	*N* = 146: 24% dysphagia.

*AYCE, About Your Child's Eating; FEES, fiberoptic evaluation of swallowing; MBQ, Mealtime Behavior Questionnaire; NG tube, nasogastric tube; OPD, oropharyngeal dysphagia; SLT, speech-language therapist; upper GI, upper gastrointestinal; VFSS, videofluoroscopic swallow study*.

*Definitions of FSD that were presented in the source articles are presented as direct quotes within quotation marks*.

*Aspiration was documented on videofluoroscopic swallow studies or fiberoptic endoscopic evaluation of swallowing; vocal cord dysfunction was assessed by laryngoscopy*.

**Figure 2:**
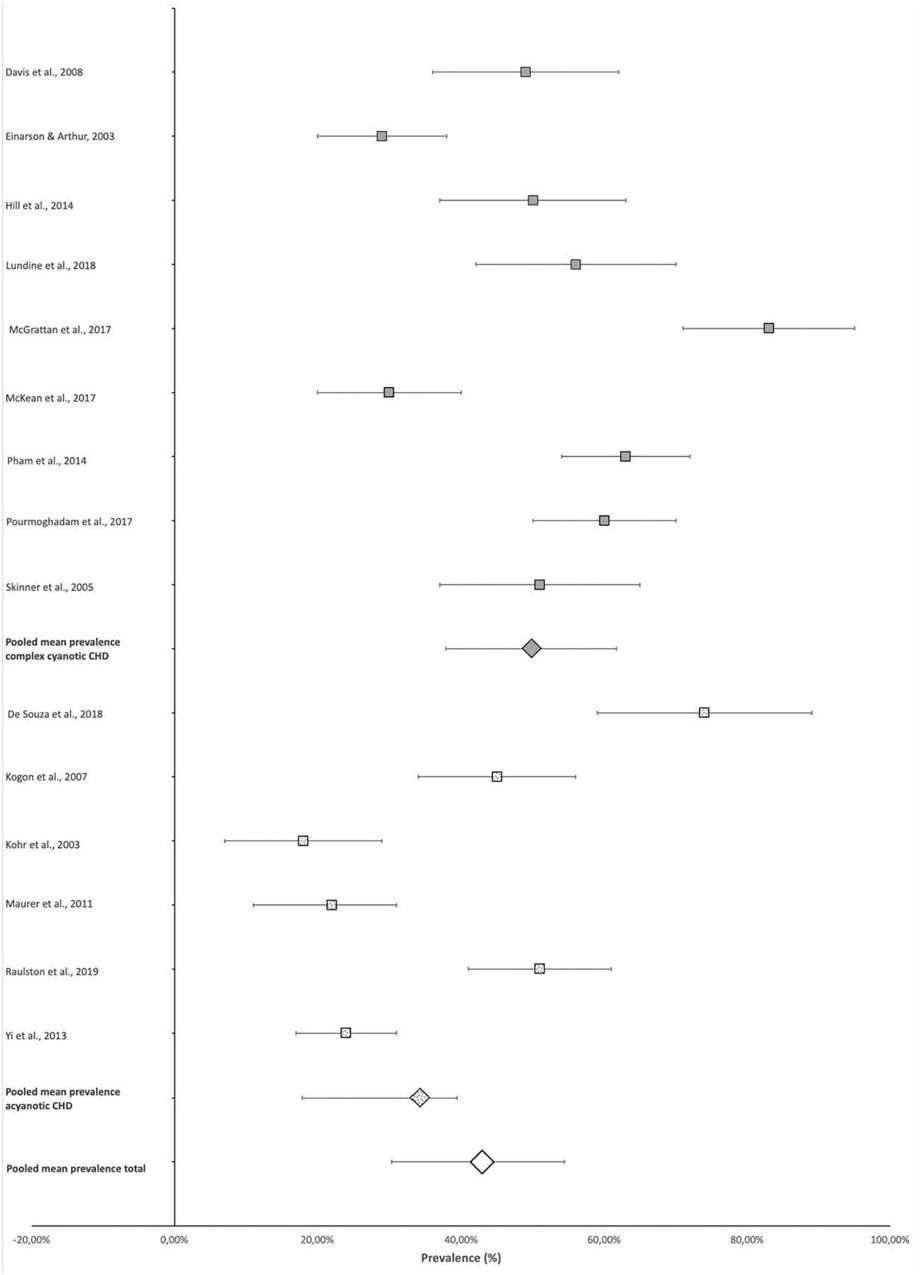
Prevalence of feeding and swallowing difficulties.

Four-hundred and seventy-seven of the total 1,107 participants included in the scoping review articles presented with some type of FSD as defined in the individual studies (Table 2), indicating an overall pooled prevalence of FSD of 42.9% (95% CI 30.4–54.4%). The prevalence of FSD reported in individual studies ranged from 18% (95% CI 7–29%) (14) to 83% (95% CI 71–95%) (17) (Figure 2).

The mean pooled prevalence of FSD in studies that included participants with more complex CHD (9, 11, 12, 15, 17–20, 22), who were likely cyanotic, was 49.9% (95% CI 37.8–61.7%) compared with the 32.5% (95% CI 20.0–43.3%) pooled prevalence of the remaining studies with predominantly acyanotic cardiac defects (10, 13, 14, 16, 21, 23). This constitutes a 15.7% (95% CI 9.9%−21.4%) difference in prevalence (*p* < 0.0001) (Figure 2).

**Figure 3:**
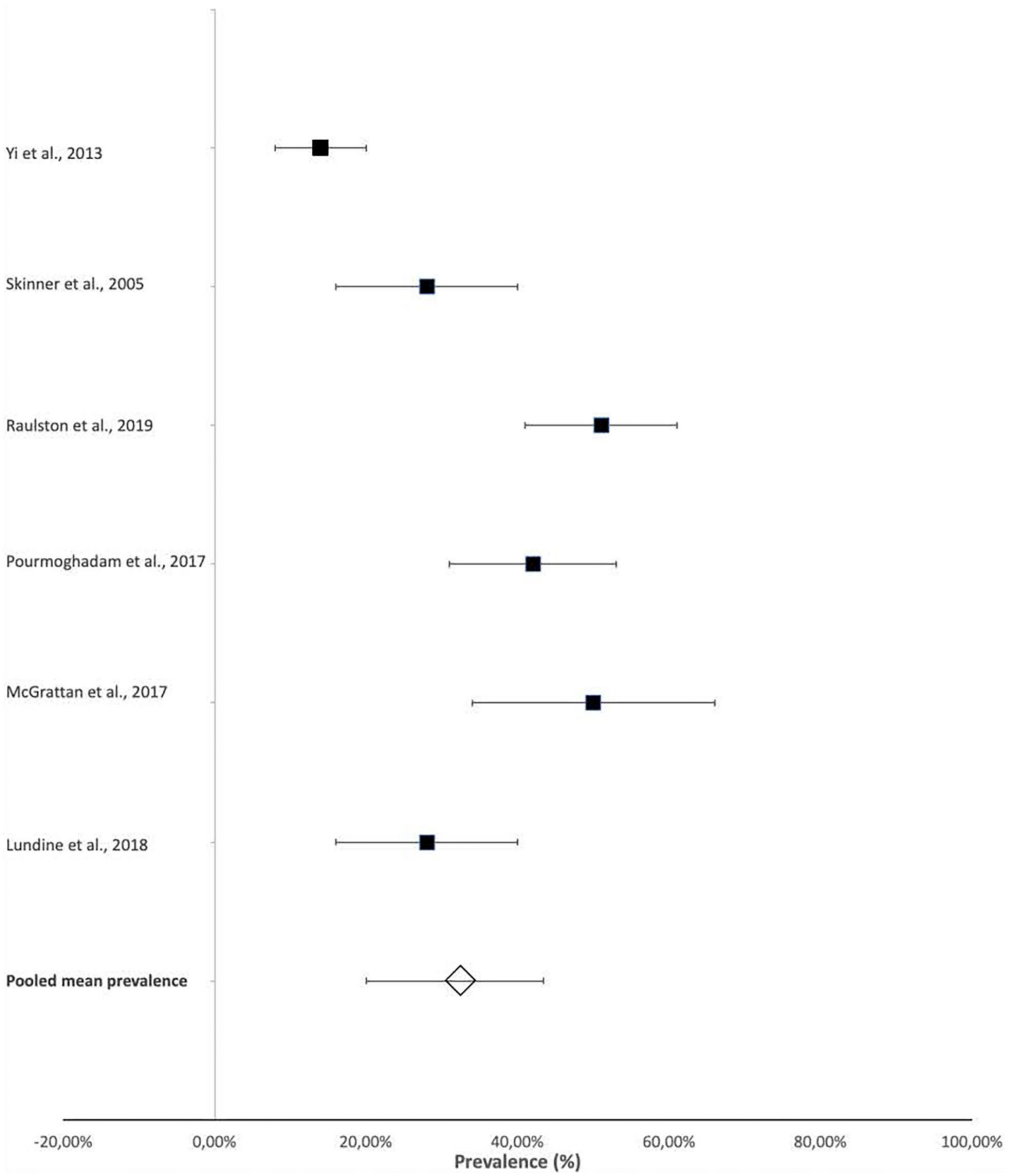
Prevalence of aspiration in infants and children with CHD. *Aspiration was documented on videofluoroscopic swallow studies or fiberoptic endoscopic evaluation of swallowing.

The prevalence of laryngeal penetration and aspiration were also reported in some studies that provided more specific details on the presenting dysphagia within their participants. This information was obtained from videofluoroscopic swallow studies (VFSS) or fiberoptic endoscopic evaluation of swallowing (FEES). The reported prevalence of aspiration ranged from 14% (95% CI 8–20%) (23) to 51% (95% CI 41–61%) (21), with a pooled mean prevalence of 32.9% (95% CI 20–43.25%) (Figure 3).

**Figure 4:**
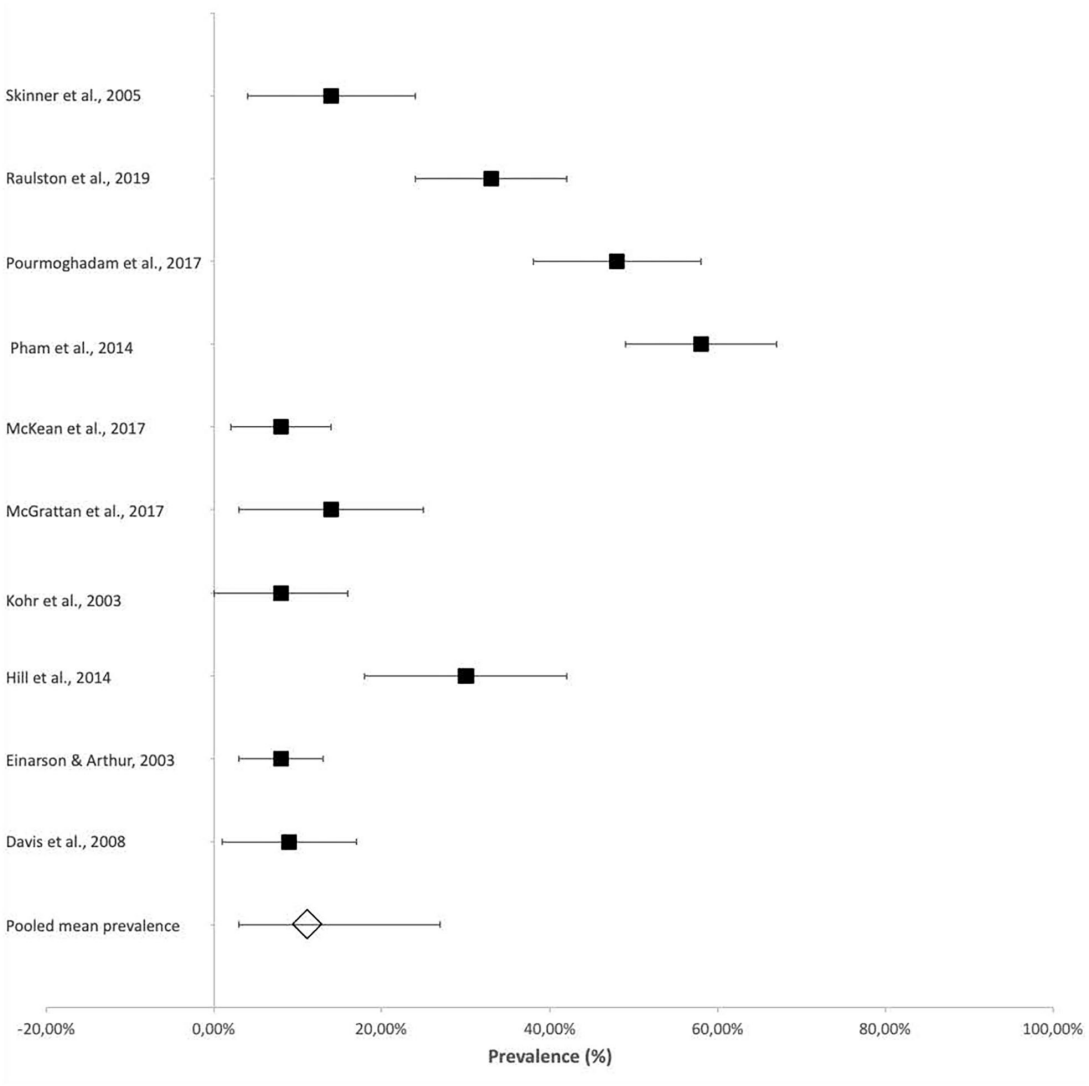
Prevalence of vocal cord dysfunction after cardiac surgery. *Vocal cord dysfunction was assessed by laryngoscopy.

Vocal cord dysfunction, as assessed by laryngoscopy, was reported in several studies with reference to the association between vocal cord function and dysphagia, particularly related to an increased risk for aspiration. The reported prevalence of vocal cord dysfunction ranged from 8% (95% CI 2–14%) (18) in patients who were followed up at 3 years of age to 57.6% (95% CI 49–67%) in neonates (Figure 4) (19).

**Figure 5:**
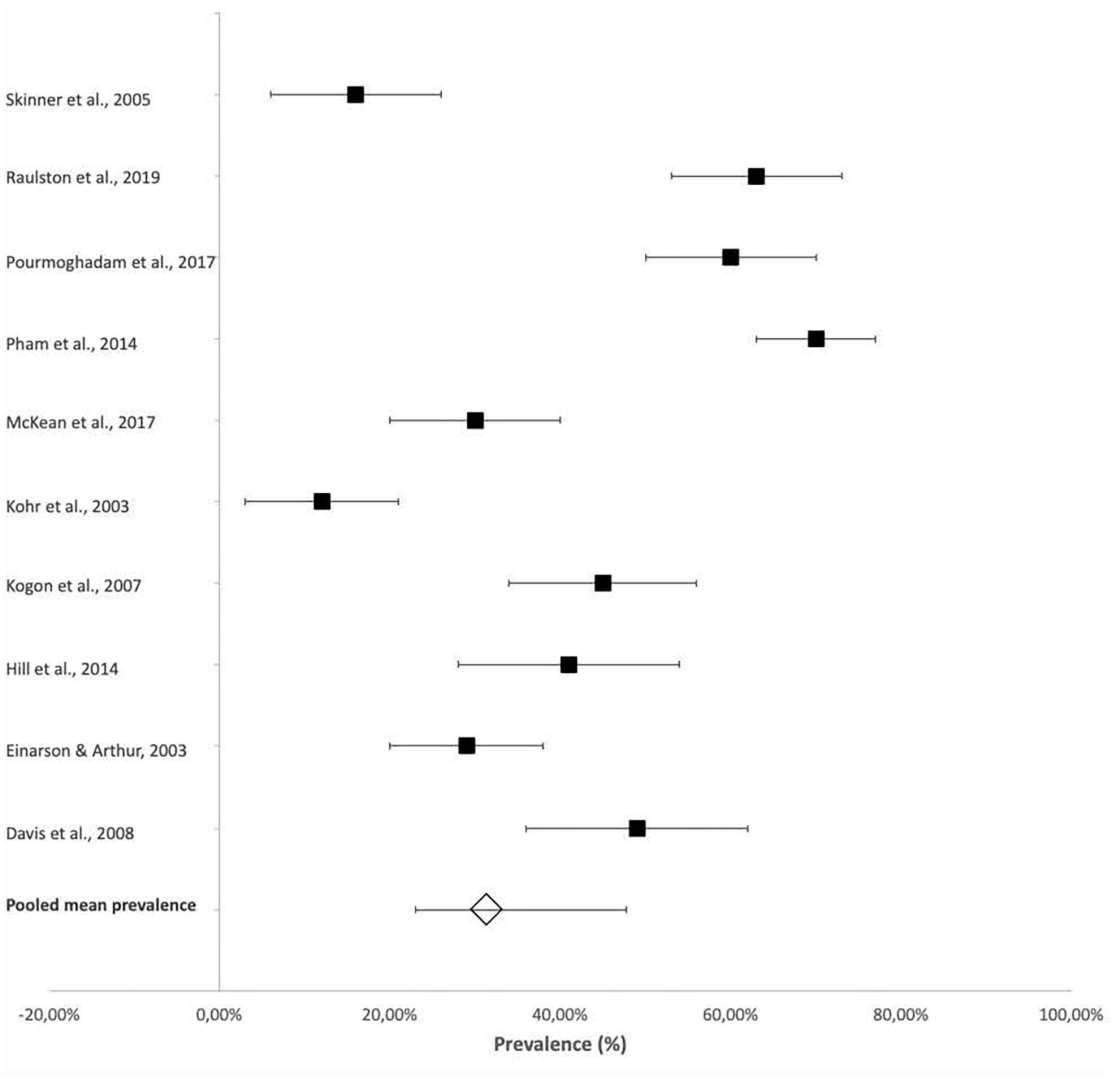
Prevalence of tube feeding reported in infants and children with CHD at and after discharge from hospital.

Tube feeding at discharge was frequently reported and was also considered a measure of FSD in some studies. A pooled mean prevalence of 31.3% (95% CI 22.9–45.3%) of tube feeding at discharge was reported across the studies (Figure 5).

## Discussion

Fifteen studies met the inclusion criteria, with the majority conducted in high-income countries within the last 10 years.

The quality of the studies was assessed using an adapted Newcastle-Ottawa Scale as most studies used retrospective research designs, with no comparison group. Most studies included representative samples, controlled for conditions other than CHD associated with FSD (possible confounders), and conducted appropriate statistical analyses. However, only one study justified the sample size used, and at least a third of the studies did not define FSD and/or did not implement a standardized clinical or instrumental assessment of FSD. There are inherent limitations in extracting information specific to FSD from studies that did not uniformly or specifically define or assess FSD. Synthesis of data from predominantly retrospective primary studies, which constitute a low evidence level, is a further limitation of this review because this study design is characterized by a substantial risk of selection, recall and other biases (24).

More than 80% of participants were recruited during infancy, with only/three of the studies reporting on participants over 2 years of age. Only one study (14) excluded preterm infants as part of their exclusion criteria. Nine studies did not report gestational age, while the remaining five studies (15, 16, 18, 19, 21) noted that preterm infants were included in their studies. While Pham et al. (19) did not report on FSD in their preterm participants separately, the remaining four studies all reported no statistically significant association between the measure of interest (e.g. FSD, aspiration, discharge with a feeding tube) and prematurity.

Most children born with critical CHDs, such as TGA, require surgical intervention during infancy, and many experience extended hospital and prolonged PICU admissions. Thus, it can be expected that the majority of FSDs will be recognized in this age group (25), with the potential to improve over time with clinical stabilization and age-related development (20, 22). Nevertheless, it is important to provide early and appropriate intervention for FSD to ameliorate the negative health consequences of this condition, and to optimize weight gain, particularly if follow-up cardiac surgery is required (26). In addition, a possible association between FSD and interstage mortality has been described, highlighting the importance of early recognition and management of FSD aimed at improving patient outcomes (22). Swallowing assessment and management should therefore form part of the standard of care for infants and children with CHDs. Those at risk should ideally be identified in the PICU for assessment as soon as appropriate (14, 19, 23).

A total of 447 participants across the included studies presented with FSD, with a pooled mean prevalence of ~43%. However, this pooled prevalence should be interpreted with caution due to the heterogeneity of the participants included in the studies. The variable definitions of FSD – and the different age ranges of participants – may account for the varied FSD prevalences reported in individual studies, ranging from 18% (14) to 83% (17). The study that reported the lowest prevalence of 18% included participants with a wide age range from birth to 17 years of age, while the two studies with the highest reported prevalences of 83% (17) and 74% (10) were conducted with neonates soon after their stage 1 palliative surgery (Norwood or Hybrid procedure) (17) and infants <6 weeks post-operatively (10). This may suggest that FSDs are of more concern in infancy and may resolve or change over time. However, this requires further research. In addition, a statistically significant difference was noted when comparing the pooled prevalence of the studies that included participants with more complex CHDs, who were likely cyanotic, to the pooled prevalence of the remaining studies, suggesting that FSDs are more likely to occur in infants and children with complex CHDs. These findings require further research.

Most studies conducted with infants reported on swallowing safety and efficiency. However, the difficulties reported by 50% of parents in an older cohort (2–6 years old) were mainly associated with negative feeding behaviors such as “mealtime aggression,” “child's resistance to eating” and “parental aversion to mealtimes” (12). These results suggest that feeding difficulties may persist into childhood and that early experience of swallowing disorders such as aspiration, may result in negative associations with feeding. Furthermore, findings suggest the need for high oral intake during the interstage period of home feeding may also be linked to increased negative mealtime experiences and behaviors (12). Early intervention to improve FSD and mealtime experiences may therefore also impact the long-term feeding outcomes for infants with CHD and their families.

Many of the included studies did not clearly describe the method of feeding and swallowing assessment used. Clinical swallowing assessments, typically conducted by a speech-language therapist, were only implemented in six of the 15 studies (9, 10, 14, 19–21), as were instrumental assessments (VFSS) (15, 17, 18, 21–23). This suggests that many studies did not determine FSD based on swallowing but rather on surrogate indicators such as tube feeding at discharge. Tube feeding alone is not specific to FSD, considering there are multiple indications for this intervention. Growth failure is common in infants with CHD and increases the risk of post-operative complications. Therefore, optimizing growth is typically part of the management of infants with CHD and may require tube feeding (26, 27). The results from studies that used clinical or instrumental swallowing assessments could be considered more reliable indicators of the prevalence of FSD. Other indicators, such as tube feeding at discharge, are non-specific descriptions of inadequate oral feeding often related to other factors such as the child's overall medical condition rather than a specific swallowing difficulty. If a clinical or instrumental assessment of feeding and swallowing was not conducted, this might suggest that participants did not receive speech-language therapy intervention aimed at optimizing feeding or swallowing. Improved health and developmental outcomes are associated with intervention for FSD and should therefore form part of the standard of care for infants and children with CHD who have FSD.

Kohr et al. (14) and De Souza et al. (10) reported FSDs based on clinical feeding and swallowing assessments conducted by a speech-language therapist with all their participants. The prevalence reported by these studies was 18 and 74%, respectively. This wide range of reported prevalence of FSD may reflect the lack of a standardized clinical assessment protocol. Studies that diagnosed dysphagia with instrumental assessments, such as VFSS or FEES, reported FSD in 52% (22) and 56% (15) of their infant participants, and 72% (17) in neonates. These higher prevalence rates might be related to the age of the participants, or they may reflect selection bias, i.e., patients referred for instrumental assessment may have clear clinical signs of swallowing difficulties or aspiration and therefore be more likely to present with FSD than those who do not present with clinical signs of FSD. However, these particular studies all followed a standard hospital protocol where all patients were assessed instrumentally post-surgery, thereby minimizing the possibility of selection bias.

Clinical signs or symptoms such as coughing or desaturation with feeds are considered possible indicators of swallowing difficulty or aspiration. However, Raulston et al. (21) reported that 27% of their participants who aspirated were asymptomatic on clinical assessment, and Lundine et al. (15) noted that bedside clinical evaluations of swallowing were only 73% sensitive in identifying aspiration. A systematic review of the accuracy of the clinical swallowing evaluation in pediatrics reported reduced sensitivity and specificity of the clinical assessment in identifying aspiration, particularly with consistencies other than thin liquids (28). In addition, silent aspiration may be missed in the clinical assessment. Therefore, Skinner et al. (22) suggested that routine instrumental assessment of vocal cord function and swallowing safety should be considered to reduce interstage mortality.

Aspiration, determined by instrumental assessment (VFSS or FEES), ranged from 8% (9) to 51% (21). This wide range of reported aspiration is likely related to the age of the participants at the time of assessment, methodological differences between studies (e.g., whether all participants were assessed with VFSS or FEES, or only those who demonstrated clinical symptoms of dysphagia and aspiration) and the particular CHD or surgical repair conducted (9, 15, 17, 20–23). Notably, though, Skinner et al. (22) compared participants who had a Norwood procedure to those who had a biventricular repair and did not find a statistically significant difference in FSD or aspiration rates.

Vocal cord dysfunction is frequently cited as the cause for increased risk of aspiration in patients post cardiac surgery. However, a statistically significant association between vocal cord dysfunction and aspiration was not reported by McGrattan et al. (17) or Skinner et al. (22). Raulston et al. (21) reported that vocal cord dysfunction was a significant risk factor for aspiration in their study, with 72% of participants with vocal cord dysfunction demonstrating aspiration. However, vocal cord dysfunction accounted for less than half of aspiration cases reported in their study, with 53% of participants who aspirated demonstrating normal vocal cord function. In addition, the study itself was not powered for a risk factor analysis. This finding is similar to McGrattan et al. (17), who reported that 48% of participants with normal laryngeal function aspirated. These findings suggest that swallowing difficulties presenting in infants post cardiac repair surgery may be related to other potential causes, such as neurologic and respiratory morbidity, and may not be specific to laryngeal dysfunction (17, 22). A protocol that includes post-operative assessment of swallowing function prior to discharge from hospital was suggested in some studies included in the review (15, 17, 21, 22) and should be considered in future research and the development of clinical guidelines.

The need for tube feeding at discharge was reported as a measure of feeding dysfunction in some studies (9, 11–13, 16, 18–20, 23). Although many infants appeared to require tube feeding when discharged home, those requiring long-term tube feeding reduced over time. Only 10% of the infants who required gastrostomy feeding initially were still feeding via a gastrostomy at the 3-year follow up (20), suggesting that feeding improved over time. Tube feeding at initial discharge may also be related to the need for interstage weight gain, and therefore the need to maximize caloric intake, possibly with continuous or overnight tube feeds (27).

The lower prevalence rate reported in studies with a wider age range also suggests that FSDs may improve over time. Nevertheless, the potential negative consequences of FSD, including aspiration, reduced oral intake, longer hospital stays, and associated negative feeding and mealtime experience, support the need for early referral for assessment and management of FSD. The timing of the swallowing assessment will vary depending on the patient's medical stability and oxygen requirements (29) but should be conducted as soon after extubation as possible (14). FSD are one of the most common morbidities following cardiac surgery noted by both families and clinicians, have an impact on quality of life, and are associated with increased PICU and hospital stays, as well as significantly higher hospital costs (30). In addition, with the knowledge that FSD in this population changes over time and may still be present years after surgery (16), and the current focus on long-term follow-up and outcomes of patients admitted to the PICU (31), ongoing surveillance of swallowing function and FSD outcomes should form part of the long-term management of infants and children with CHD.

## Conclusion

The scoping review demonstrates that FSDs are common in infants and children with CHD, with over 50% prevalence reported in infancy in most studies. This constitutes many patients with FSD who face risks to their health and well-being. Early referral for assessment and management should therefore be considered in infants with CHD as standard of care. While it may not always be appropriate or possible to assess swallowing while the patient is still in the PICU, it is recommended that the need for a swallowing assessment be noted on handover from PICU and a requirement before discharge from hospital, as swallowing difficulties are associated with increased morbidity and mortality (15, 17, 21, 22). This practice was not apparent in many studies, where feeding and swallowing were not specifically assessed, or assessed according to a standard protocol. Although FSD intervention was not specifically described, this finding suggests that targeted speech-language therapy intervention was also suboptimal.

The prevalence of FSD in older children – as well as long-term outcomes of infantile FSD – was poorly described and requires further investigation. Lack of information regarding the gestational age in most studies, the inclusion of preterm participants, as well as infants and children with other comorbidities associated with FSD, are a limitation and future studies should have clearer selection criteria or report on FSD in these groups separately.

The lack of a global definition and gold standard clinical diagnostic assessment of FSD limits the ability to directly compare studies and constitutes a clinical limitation. A consensus definition for pediatric feeding disorders was recently proposed by Goday et al. (32), which may address this challenge in future research.

This scoping review summarizes the available information on the prevalence and nature of FSD in infants and children with CHD and highlights several critical gaps in the literature. Studies from low-middle income countries are limited and urgently needed, together with long-term studies of FSD trajectories and outcomes. There is a need for a globally accepted, standardized definition of FSD to allow comparison across centers, and development of standard protocols or clinical guidelines for the routine assessment of feeding and swallowing in infants with CHD is required. An instrumental assessment is particularly needed because of the potential for silent aspiration and the possible contribution of FSD to interstage mortality.

## Data Availability Statement

The original contributions presented in the study are included in the article/Supplementary Material, further inquiries can be directed to the corresponding author/s.

## Author Contributions

VN, LZ, and BM contributed to the conceptualization of the study which was undertaken as part of VN's PhD under the supervision of BM and LZ. VN and KM were involved in data collection and analysis. VN wrote the first draft of the manuscript and thereafter all authors revised the manuscript. All authors contributed to the article and approved the submitted version.

## Conflict of Interest

The authors declare that the research was conducted in the absence of any commercial or financial relationships that could be construed as a potential conflict of interest.

## Publisher's Note

All claims expressed in this article are solely those of the authors and do not necessarily represent those of their affiliated organizations, or those of the publisher, the editors and the reviewers. Any product that may be evaluated in this article, or claim that may be made by its manufacturer, is not guaranteed or endorsed by the publisher.
