# Supplementary material for: Prevalence of Feeding and Swallowing Disorders in Congenital Heart Disease: A Scoping Review
Source: Front Pediatr. 2022 Apr 5;10:843023. doi: 10.3389/fped.2022.843023 (PMC9016225; doi:10.3389/fped.2022.843023)
Supplement: Supplementary file 1 [file Data_Sheet_1.docx]

Supplementary Material

**Supplementary Table 1: Example of search strategy**

| **DATABASE: PUBMED** | | | |
| --- | --- | --- | --- |
|  | | **Search terms** | **Number of articles** |
| #1 | [MeSH] | Deglutition | 50777 |
| #2 | Key terms | Dysphagia OR deglutition disorder OR deglutition disorders OR swallowing disorder OR swallowing disorders OR swallowing difficulty OR swallowing difficulties OR feeding behavior OR feeding behaviour OR feeding disorder OR feeding disorders OR feeding difficulty OR feeding difficulties | 278241 |
| #3 |  | #1 OR #2 | 278241 |
| #4 | [MeSH] | Heart Defects, Congenital | 146597 |
| #5 | [MeSH] | Heart Ventricles/abnormalities | 4353 |
| #6 | Key terms | Congenital heart defects OR congenital heart diseases OR congenital heart disease OR congenital cardiovascular abnormalities OR congenital cardiovascular diseases OR congenital cardiovascular disease OR heart ventricle abnormality OR heart ventricle abnormalities | 194291 |
| #7 |  | #4 OR #5 OR #6 | 194291 |
| #8 | [MeSH] | Infant | 1105848 |
| #9 | [MeSH] | Child | 1846671 |
| #10 | [MeSH] | Pediatrics | 55882 |
| #11 | Key terms | Child OR children OR infant OR infants OR newborn OR neonate OR neonates OR paediatric OR pediatric | 3267362 |
| #12 |  | #8 OR #9 OR #10 OR #11 | 3267362 |
| #13 | [MeSH] | Prevalence | 273993 |
| #14 | [MeSH] | Epidemiology | 25993 |
| #15 | Key terms | associated OR association OR associations OR burden OR case-control OR cohort OR correlation OR correlates OR course OR cross-sectional OR determinant OR epidemiology OR epidemiological OR epidemiologic OR follow up OR follow-up OR followup OR frequency OR incidence OR interview OR likelihood ratio OR observational studies OR observational study OR occur OR occurrence OR odds ratios OR predict OR predictor OR prediction OR present OR presentation OR prevalence OR prevalent OR probability OR prognosis OR prognostic OR proportion OR prospective OR questionnaire OR rate OR retrospective OR risk OR risks OR self-report OR statistic OR surveillance OR survey | 13705268 |
| #16 |  | #13 OR # 14 OR # 15 | 13705268 |
| #17 |  | #3 AND #7 AND #12 AND #16 | 535 |
|  |  | Added limitations:  Published from 1995 to current (2020)  Humans  English  Child (0 – 18) | 368 |
| **Final articles identified and imported from Pubmed** | | | **368** |

**Supplementary Table 2: Adapted NOS Quality score for included articles**

| **Study/Reference** | **Selection** | | | | **Comparability** | **Outcome** | | **Total *** |
| --- | --- | --- | --- | --- | --- | --- | --- | --- |
|  | **Representativeness of the sample** | **Sample size:**  **justified & satisfactory** | **Ascertainment of exposure: FSD clearly defined** | **Ascertainment of exposure:**  **FSD assessed clinically or instrumentally** | **Study controls for other conditions associated with FSD** | **Reports on assessment of outcomes** | **Statistics**  **(included measurements / *p*-values)** | **Maximum score 7** |
| **Davis et al. (9)** | * | - | - | - | * | - | * | 3/7 |
| **De Souza et al. (10)** | * | - | * | * | * | * | * | 6/7 |
| **Einarson & Arthur (11)** | * | * | * | - | * | - | * | 5/7 |
| **Hill et al. (12)** | - | - | * | - | - | * | * | 3/7 |
| **Kogon et al. (13)** | * | - | * | - | - | - | * | 3/7 |
| **Kohr et al. (14)** | * | - | * | * | * | * | * | 6/7 |
| **Lundine et al. (15)** | * | - | * | * | * | * | * | 6/7 |
| **Maurer et al. (16)** | * | - | * | - | - | * | * | 4/7 |
| **McGrattan et al. (17)** | * | - | - | * | * | * | * | 5/7 |
| **McKean et al. (18)** | * | - | * | - | - | * | * | 4/7 |
| **Pham et al. (19)** | * | - | * | * | - | * | * | 5/7 |
| **Pourmoghadam et al. (20)** | * | - | - | * | - | * | * | 4/7 |
| **Raulston et al. (21)** | - | - | - | * | - | * | * | 3/7 |
| **Skinner et al. (22)** | * | - | - | * | Did not control for other conditions but analyzed separately * | * | * | 5/7 |
| **Yi et al. (23)** | * | - | * | * | * | * | * | 6/7 |
